# Principles of temporal association cortex organisation as revealed by connectivity gradients

**DOI:** 10.1007/s00429-020-02047-0

**Published:** 2020-03-10

**Authors:** Guilherme Blazquez Freches, Koen V. Haak, Katherine L. Bryant, Matthias Schurz, Christian F. Beckmann, Rogier B. Mars

**Affiliations:** 1 Donders Institute for Brain, Cognition and Behaviour, Radboud University Medical Center, Nijmegen, The Netherlands; 2 Donders Institute for Brain, Cognition and Behaviour, Radboud University, Nijmegen, The Netherlands; 3 Wellcome Centre for Integrative Neuroimaging, Centre for Functional MRI of the Brain (FMRIB), Nuffield Department of Clinical Neurosciences, John Radcliffe Hospital, University of Oxford, Oxford, United Kingdom

**Keywords:** Tractography, Connectivity gradients, Laplacian eigenmapping, Temporal lobe

## Abstract

To establish the link between structure and function of any large area of the neocortex, it is helpful to identify its principles of organisation. One way to establish such principles is to investigate how differences in whole-brain connectivity are structured across the area. Here, we use Laplacian eigenmaps on diffusion MRI tractography data to investigate the organisational principles of the human temporal association cortex. We identify three overlapping gradients of connectivity that are, for the most part, consistent across hemispheres. The first gradient reveals an inferior–superior organisation of predominantly longitudinal tracts and separates visual and auditory unimodal and multimodal cortices. The second gradient radiates outward from the posterior middle temporal cortex with the arcuate fascicle as a distinguishing feature; the third gradient is concentrated in the anterior temporal lobe and emanates towards its posterior end. We describe the functional relevance of each of these gradients through the meta-analysis of data from the neuroimaging literature. Together, these results unravel the overlapping dimensions of structural organization of the human temporal cortex and provide a framework underlying its functional multiplicity.

**Electronic supplementary material:**

The online version of this article (10.1007/s00429-020-02047-0) contains supplementary material, which is available to authorized users.

## Introduction

Understanding the relationship between brain structure and function is one of the main challenges of neuroscience (Honey et al. 2009). To establish this link, rather than solely providing maps of cortical areas and their connections, a definition of the rules that underlie their pattern—the principles of organisation—is required, such that it is possible to predict aspects of organisation and their functional consequences even if they are yet to be characterised. Well-known principles of organisation have been described at the level of specific parts of the cortex, such as the existence of several parallel processing hierarchies in frontal cortex (Kouneiher et al. 2009) and parallel processing streams connecting parietal and frontal cortex (Wise et al. 1997) that give rise to overlapping rostro-caudal and dorsal–ventral patterns of organisation (Vijayakumar et al. 2018).

Unravelling the principles of organisation of a specific region is then not only relevant from a cartographic viewpoint, but is arguably the most important step for establishing the link between structure and function; by outlining how different aspects of brain anatomy are organised, we can then interpret the computational architecture that implements cognitive function (Thivierge and Marcus 2007). For instance, the overlapping topographical principles of coding for polar angle and eccentricity in early visual cortex (Wandell et al. 2007) allow for the parallel encoding of these two dimensions using simple spatial rules (Haak et al. 2018). Another example of topography informing function can be found in the posterior parietal cortex, where functional organisation follows spatially ordered patterns, ranging from projection of environment onto the body on its rostral pole to projection of the body onto the environment on its caudal pole (Medendorp et al. 2019).

Such an understanding of principles of connectivity has not yet been reached for the temporal association cortex. The primate temporal cortex is thought to be a unique specialization and different to the lateral expansions seen in other mammalian orders (Bryant and Preuss 2018). Its constituent areas range from visual association cortex (Freedman et al. 2003) and the tonotopically organised auditory primary cortex (Humphries et al. 2010) to multimodal areas involved in a number of high-level behaviours, including categorization, semantic processing, and social cognition (Lambon Ralph et al. 2017; Schurz et al. 2014). Even within the primate order, the temporal lobe is thought to have undergone significant expansion and reorganisation, particularly in the great ape and human lineage (Bryant et al. 2019; Mars et al. 2013) where it might underlie the uniquely human specialization for language (Eichert et al. 2018). This variety of functional areas within the temporal lobe and its variability across species makes the need for an understanding of its overarching organisational principles all the more pressing. Yet, the precise link between the anatomical reorganization of the temporal lobe throughout evolution and its higher functional multiplicity remains poorly understood.

One way to examine principles of cortical organisation is to look at how specific features of organisation change across space, the so-called ‘gradients’ approach (Huntenburg et al. 2018). In this framework, connectivity—or indeed any anatomical feature—is described as gradually changing across any brain region as opposed to sharply bordered clusters of homogeneous connectivity. The direction of these gradual connectivity changes is uncovered by dimensionality reduction techniques such as Laplacian eigenmaps (Belkin and Niyogi 2003) which allow one to disentangle overlapping directions of connectivity changes (referred to as ‘modes of connectivity’), producing connection topography maps or ‘connectopic maps’ (Haak et al. 2018). For instance, Margulies and colleagues (2016) used resting state functional connectivity to show overlapping principles of organisation that describe the human neocortex as a whole, arguing that a principal mode of organisation is along an axis from primary, uni-modal areas to high-level, multi-modal areas, with a second axis corresponding to the various sensory modalities. This same approach has been used to characterise organisation of smaller parts of the brain, including the overlapping principles of visual cortex (Haak et al. 2018), parietal–frontal connectivity (Vijayakumar et al. 2019), entorhinal cortex (Navarro Schröder et al. 2015), and even subcortical structures (Marquand et al. 2017).

However, if one is interested in the relationship between brain structure and function, a more suitable starting place is diffusion MRI tractography data, which allows one to reconstruct aspects of white matter connectivity across the cerebral cortex. Using tractography data, we can search for principles of connectivity that are directly based on the major white matter fibre bundles connecting the temporal cortex with the rest of the brain, which are well described both in humans and in other primates (Bajada et al. 2017a; Catani and Thiebaut de Schotten 2008; Schmahmann and Pandya 2006). Some earlier work has used tractography data to investigate gradients of structural connectivity (Cerliani et al. 2012) including on the temporal lobe (Bajada et al. 2017b, 2019). These analyses, however, focused on global similarity between connectivity profiles, either by reducing the connectivity profiles to one dimension using spectral reordering or remapping multidimensional maps into a RGB space. Thus, the differential role of each temporal region in contributing to a multidimensional connectivity architecture may have been overlooked. Here, we address this issue by investigating whether we can find overlapping gradients of connectivity in the human temporal association cortex using tractography data.

We employ a recently proposed analysis framework extending the previously proposed Laplacian eigenmap approach to diffusion MRI tractography data (Blazquez Freches et al. 2019). We hypothesized that due to the involvement of the temporal lobe in a wide array of cognitive functions and its complex white matter tract composition (Bajada et al. 2017a), the low-dimensional embedding of its anatomical connectivity fingerprint would contain overlapping modes of connectivity (Jbabdi et al. 2013). First, we analyse the first three eigenvectors separately and propose that they constitute overlapping organisation principles of the temporal lobe. Second, we corroborate this hypothesis by associating differentiated tract projections to their corresponding connectivity mode. Finally, we apply the resultant organisational principles to meta-analysis of fMRI data to demonstrate the fine-grained characterisation of organisation derived from white-matter fibre connectivity maps on to known patterns of task activation, thus linking structural connectivity principles to their functional consequence.

## Materials and methods

### Data

Diffusion MRI and T1- and T2-weighted structural data from 42 subjects of the test–retest Human Connectome Project (HCP) (28 females, 4 left handed, aged 22–35 years) were selected (Sotiropoulos et al. 2013). Two subjects were erroneously excluded from the cohort and analysed a posteriori*—*their individual results can be found in a separate location in the online result file and show a similar pattern as the full dataset. The diffusion data consisted of isotropic 1.25-mm voxels acquired in a multi-shell sequence with *b* values 1000, 2000 and 3000 and 90 diffusion weighting directions in each shell. T1- and T2-weighted images were acquired with 0.7-mm isotropic voxels and a TR of 2.4 and 3.2 s, respectively. All data were preprocessed using the HCP’s minimal preprocessing pipeline, previously described in detail (Glasser et al. 2013; Sotiropoulos et al. 2013). In brief, the data from the different modalities were registered to one another and to MNI standard space; the T1- and T2-weighted data were used to create cortical surfaces and the Freesurfer aparc parcellation (Destrieux et al. 2010) was used to divide them into their constituent areas. Finally, the diffusion MRI data were preprocessed to create posterior distributions of fibre orientations for probabilistic tractography using FSL’s BEDPOSTX (Behrens et al. 2007; Jbabdi et al. 2012).

### Region of interest selection

A temporal cortex region-of-interest (ROI) was created on the cortical surface of each hemisphere by merging all individual temporal subregions (superior, middle, and inferior temporal gyri, banks of the superior temporal sulcus, fusiform gyrus, transverse temporal gyrus and temporal pole) from the aparc segmentation (Destrieux et al. 2010) and keeping those vertices that were assigned to any of the temporal subregions in at least 95% of the subjects. Entorhinal and parahippocampal cortices were not included as they have different architectonic characteristics from the rest of the temporal lobe (Zilles et al. 2002) and the focus of the present study was on the temporal association cortex.

### Probabilistic tractography and Laplacian eigenmaps

Probabilistic tractography was performed in each subject by seeding from the group ROI at the mid-thickness surface level to the ipsilateral hemisphere. Self-connectivity effects were mitigated by warping the group mask in each individual surface to each individual subject volume space using the workbench command ‘metric-to-volume-mapping’ and keeping those voxels from being considered in the output connectivity matrix. Stop masks were placed at the pial surface and at the mid-sagittal plane so that streamlines would not leave the brain, cross hemispheres or cross adjacent gyri. FSL’s PROBTRACKX was used with the following settings: 10,000 streamlines per voxel, maximum path length of 2000 steps, step size of 0.5 mm, and the ‘matrix2’ mode. This generated a *seed* × *hemisphere* visitation count matrix that corresponded to the tractographic connectivity profile for each temporal ROI vertex.

To compute the between-vertex similarity between seed vertices, a similarity function was applied to the matrix containing every temporal ROI vertex’s connectivity profile. In this pipeline, the *η*^2^ coefficient was chosen (Cohen et al. 2008). The similarity matrix was then transformed into a weighted graph by means of a *k*-nearest neighbours approach with the number of neighbours being the minimum necessary so that the resulting graph only contained one connected component.

To limit our group analysis to the minimum common number of dimensions across all subjects, the dimensionality of each individual network graph (at the level of the graph adjacency matrix) was estimated using maximum likelihood estimation (MLE) of intrinsic dimensionality (Levina and Bickel 2004). The minimum common subset across subjects was found to be three, meaning that the MLE algorithm estimated this number to be the minimum number of dimensions on any given subject.

The selected regions’ Laplacian eigenmaps were then obtained by performing the generalised eigen decomposition of the graph Laplacian, after discarding the first eigenvector (0-valued eigenvalue) (von Luxburg 2007). In this study, the three eigenvectors (normalised between one and ten and called connectopic maps) associated with first three non-zero eigenvalues were investigated. All individual gradients were aligned to a reference subject by computing the Pearson correlation of the reference gradient to the target gradient. The target gradient would get flipped around 5.5 (the midpoint between one and ten) if the correlation was lower than 0.75.

Group-level connectopic maps were obtained by averaging all subjects’ individual connectopic maps within a dataset (test or retest).

### Projection images and tract projections

To investigate how the connectopic topographies for a given grey matter area are related to connections with underlying white matter, we created tract projection images for each eigenvector. First, and for every subject, we created a projection skeleton in volume space, showing for each voxel in the target hemisphere how often a streamline from the seed had reached it. This projection skeleton was created by thresholding (such that only voxels visited by at least 1% of streamlines or a given seed vertex were considered) and binarizing the matrix resulting from FSL’s PROBTRACKX. Second, we populated each voxel of the projection skeleton with the weighted average connectopy value of the top three vertices whose streamlines hit that target voxel the most, thereby producing the projection images. Each connectopic map will have one projection image associated with it.

Every projection image was multiplied with individualised thresholded white matter tract tractograms obtained using FSL’s XTract (Warrington et al. in review.)—a toolbox and library of standardised tractography protocols devised for the robust automated extraction of white matter tracts—following the tract definitions described in Mars et al. (2018) to separate the contributions of each white matter tract to the overall connectome. We refer to the resulting images as tract projections. In the temporal lobe, the following tracts were considered: inferior longitudinal fasciculus (ILF), arcuate fasciculus (AF), acoustic radiation (AR), inferior fronto-occipital fasciculus (IFOF), middle longitudinal fasciculus (MdLF) and the uncinate fasciculus (UF).

### Cross-subject and cross-session subject connectopic map correlation

To assess cross-subject and cross-session reliability of the connectopic maps, the intra-class correlation coefficient (ICC) case 2.1 (Shrout and Fleiss 1979) was used with *k* = 2 for both cross-subject and cross-session ICC. Both cross-subject and cross-session ICC were defined as the bootstrapped 95% confidence interval of the means of their respective definitions. The bootstrap was made with 10,000 samples.

### Link to function

While connectopic maps uncover the multidimensional gradual change of connectivity patterns within the temporal lobe they do not assign them any functional relevance. The link between anatomical connectivity modes to their functional importance was investigated by dividing each group connectopic map into deciles, and decoding each decile with Neurovault (Gorgolewski et al. 2015). This approach consists of spatially correlating each decile of each connectopic map with thousands of fMRI studies present in the Neurosynth database (Yarkoni et al. 2011). The top three non-anatomical terms (corresponding to the terms whose maps had the highest spatial Pearson correlations to our connectopic map deciles) for each decile were kept, and redundant terms such as ‘object’ and ‘objects’ were only considered once. For simplicity, we only show positive correlations found by decoding as negative correlations not directly inform us about processed linked to gradient maps. The reported values and terms are the ones measured at the time the maps were posted on Neurovault (see “Open science statement”).

### Open science statement

The code to perform tractography is part of FSL (www.fmrib.ox.ac.uk/fsl), the code to perform Laplacian eigenmapping and tract projection is part of the MR Comparative Anatomy Toolbox (MrCat; www.neuroecologylab.org). Data are available as part of the Human Connectome Project (www.humanconnectome.org). Results files will be made available as part of a Data Sharing Collection on the Donders Repository (see link at www.neuroecologylab.org) and on https://github.com/gfreches/temporal-lobe-organisation. Neurovault deciles can be found in https://neurovault.org/collections/5575/ (for left hemisphere) and https://neurovault.org/collections/5594/ (for right hemisphere).

## Results

### Three distinct and overlapping connectivity gradients coexist within the temporal lobe

We performed probabilistic tractography from the temporal lobe cortical surface towards the ipsilateral hemisphere and submitted the resulting tractography matrix for Laplacian eigenmapping. Individual subjects’ maps were averaged together to produce group maps reflecting the global modes of connectivity of the temporal lobe. An MLE estimate of intrinsic dimensionality indicated three distinct, overlapping modes of connectivity.

**Fig. 1:**
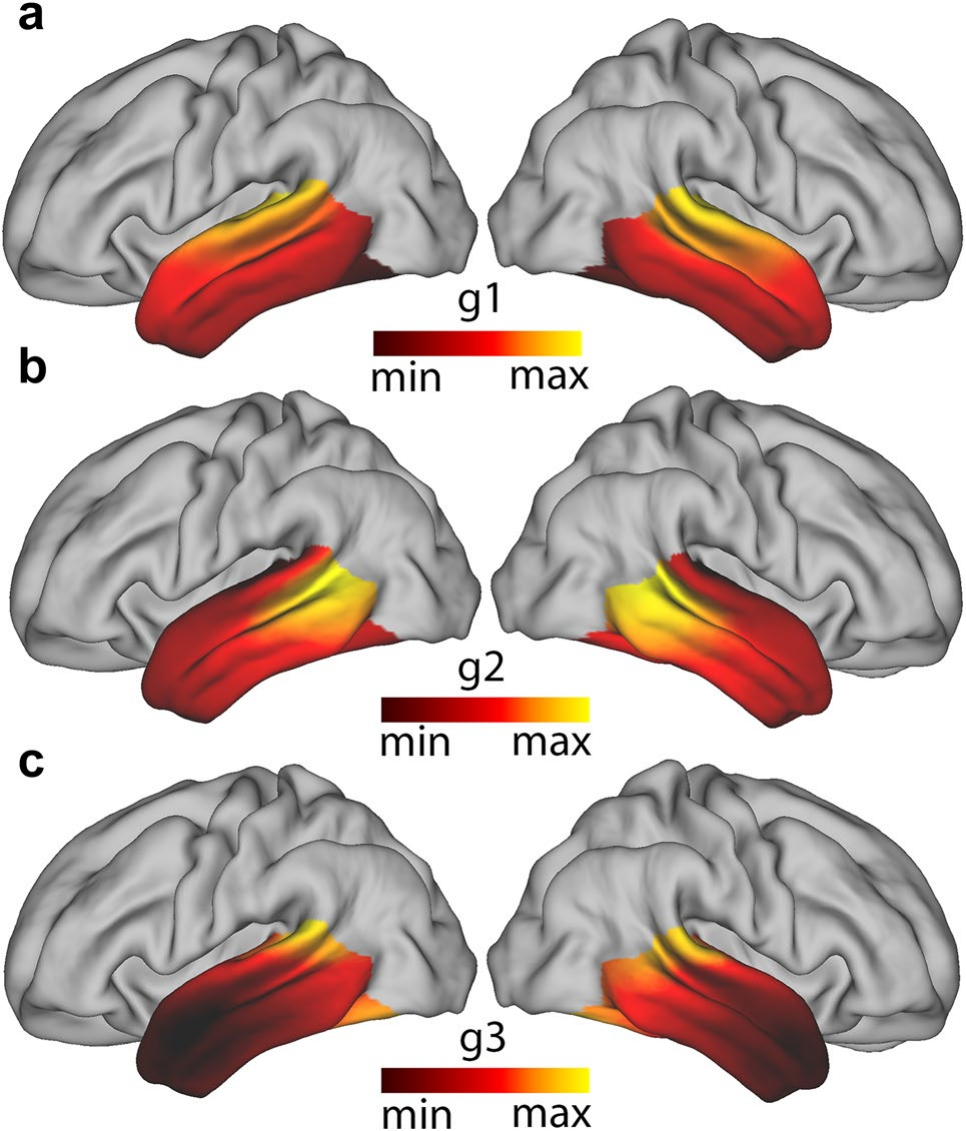
Group-averaged connectivity modes projected on a mid-thickness surface: **a** Group-averaged dominant mode of connectivity (g1), **b** group-averaged second dominant mode of connectivity (g2), **c** group-averaged third dominant mode of connectivity (g3) *Left* left hemisphere, *Right* right hemisphere. Similar colours represent similar gradient values

The first mode of connectivity (Fig. 1a) showed an inferior–superior gradient. Similar values are present along the rostro-caudal axis of each lateral gyrus. The second mode of connectivity map (Fig. 1b) is radial, emanating from the posterior medial temporal sulcus (pMTS), suggesting this area of the temporal lobe has a very distinct connectivity signature from the rest of its constituents. The third mode (Fig. 1c) is also radial but emanating from the anterior part of the temporal cortex, including the temporal pole. These three modes of connectivity were obtained in both hemispheres, even though the analyses were run independently.

Previous studies looking at gradual connectivity changes within a region of the brain have also found that maps are subject-specific and can provide some understanding of phenomena such as lateralisation (Blazquez Freches et al. 2019; Haak et al. 2018; Marquand et al. 2017). For this reason, the intraclass correlation coefficient (ICC) (Shrout and Fleiss 1979) was used to evaluate the robustness and consistency of the temporal connectivity gradient maps (Table 1). This revealed that the between-session ICC is always higher than between-subject ICC, indicating that individual connectopies retain subject-specific information when the said subject is scanned twice.

**Table 1:** Reproducibility of connectopic mapping at the single-subject level

Comparison	Temporal (g1)	Temporal (g2)	Temporal (g3)
Between sessions	L—0.778 [0.689–0.836]	L—0.749 [0.664–0.806]	L—0.607 [0.502–0.685]
	R—0.849 [0.810–0.849]	R—0.651 [0.536–0.722]	R—0.422 [0.277–0.533]
Between subjects			
Session 1	L—0.758 [0.745–0.771]	L—0,620 [0,606–0.634]	L—0.496 [0.480–0.512]
	R—0.783 [0.777–0.794]	R—0.497 [0.478–0.515]	R—0.319 [0.296–0.341]
Session 2	L—0.764 [0.751–0.776]	L—0,661 [0,649–0.672]	L—0.500 [0.483–0.518]
	R—0.808 [0.799–0.816]	R—0,571 [0.556–0.585]	R—0.430 [0.410–0.450]

Reported values represent the average intra-class correlation coefficient across same subject pairs in different sessions (between sessions) or different subject pairs in the same session (between subjects). Values between square brackets indicate the lower and upper bounds of the bootstrapped 95% confidence interval with 10,000 samples, respectively

*L* left hemisphere gradient, *R *right hemisphere gradient

Both between-subject and between-session ICC drop as the order of the connectivity mode increases, which is in line with the drop in variance explained as higher order Laplacian eigenvectors are explored (von Luxburg 2007). A hemispheric asymmetry effect is also visible, in which the between-subject ICC is higher in the right hemisphere in the first connectivity mode maps (g1), while the left hemisphere has higher ICC on the second (g2) and third (g3) connectivity mode gradient maps. Together, these findings suggest a differential involvement of lateralised tracts in different connectivity modes.

### Tract projections reveal how specific white matter tracts shape connectivity gradients

**Fig. 2:**
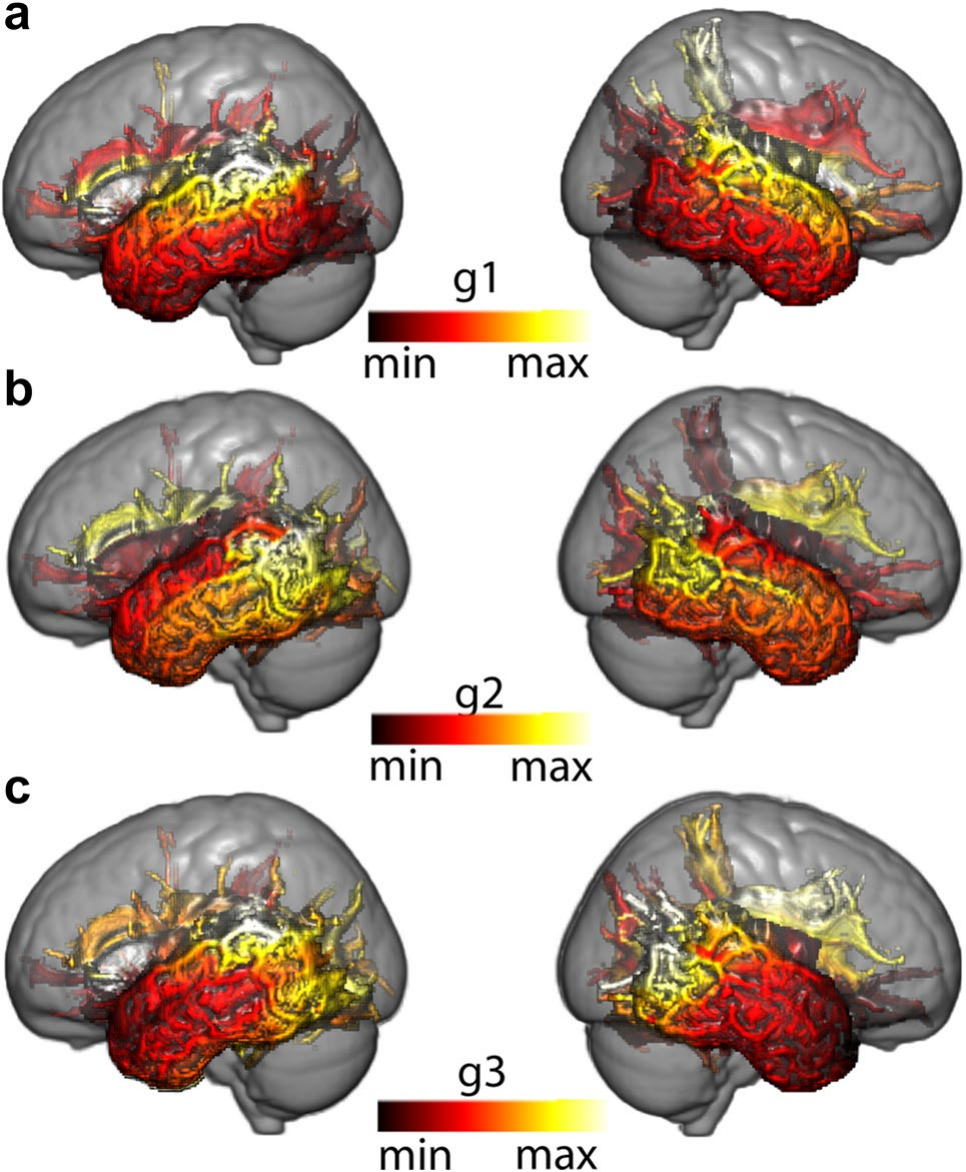
Projection images of a representative subject—103818: **a** Projection image of the dominant mode of connectivity (g1), **b** projection image of the second dominant mode of connectivity (g2), **c** projection image of the third dominant mode of connectivity (g3), *Left* left hemisphere, *Right* right hemisphere. The scale is normalised between 1 and 10 and similar colours represent similar gradient values along the represented connectivity mode

**Fig. 3:**
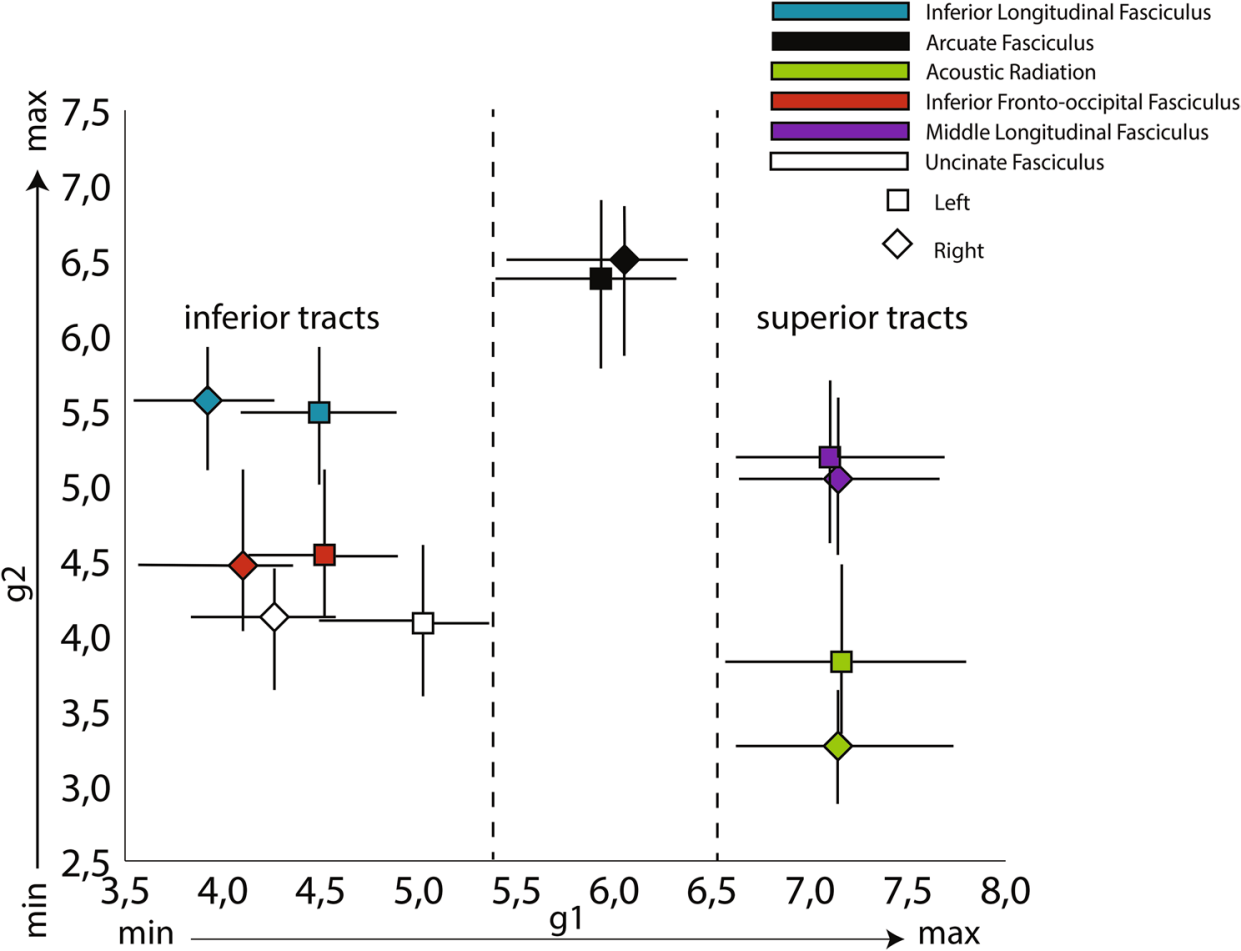
Projection image. Squares (left hemisphere) and diamonds (right hemisphere) represent average values of the temporal lobe’s relevant tract projections along the first two gradients. Dashes represent the bootstrapped 95% confidence interval of the mean. *X*-axis—value along the projection image of the first mode of connectivity (g1; from interior to superior positions); *Y*-axis—value along the projection image of the second mode of connectivity (g2; radiates from the AF).

**Fig. 4:**
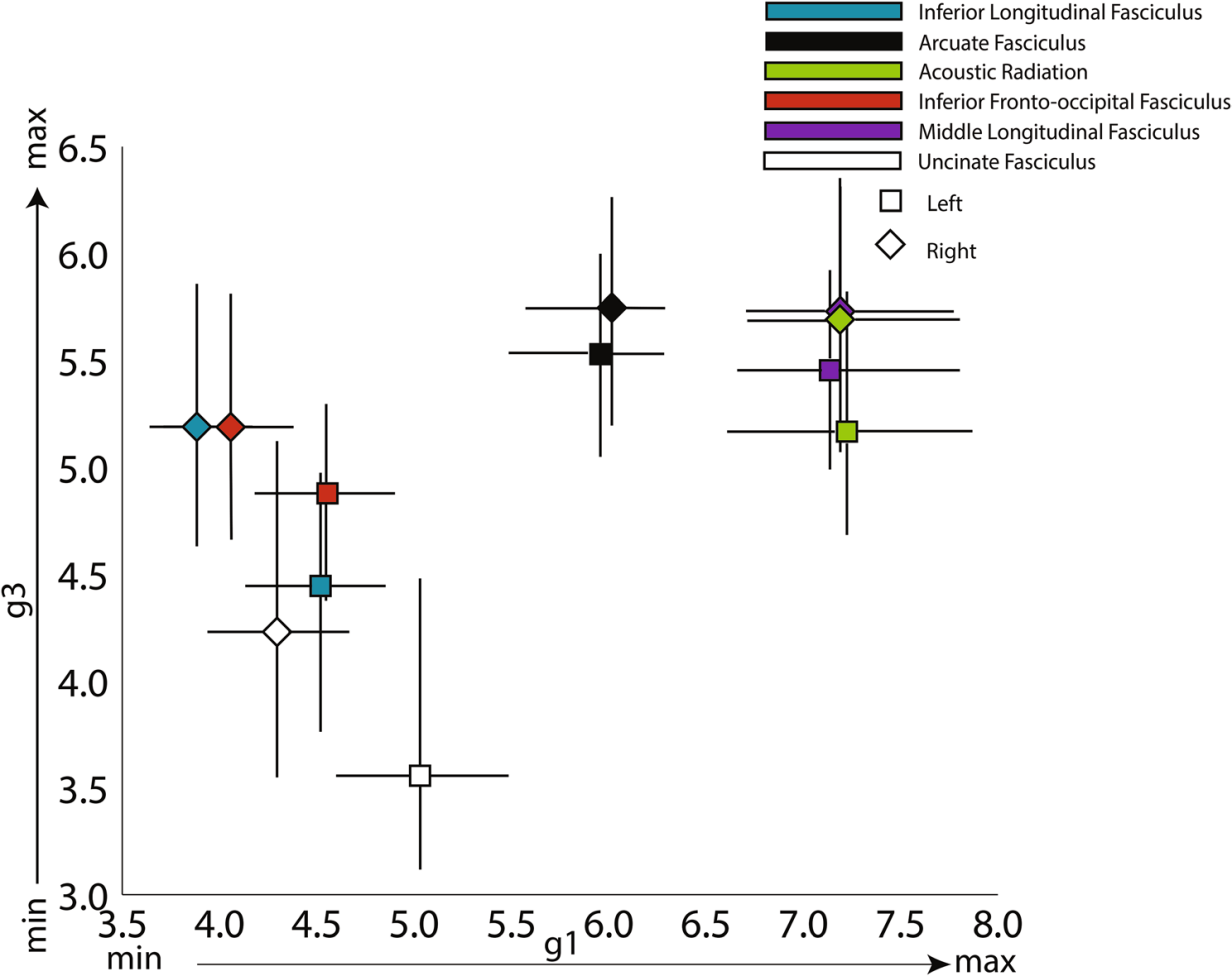
Projection image. Squares (left hemisphere) and diamonds (right hemisphere) represent average values of the temporal lobe’s relevant tract projections along the first and third gradients. Dashes represent the bootstrapped 95% confidence interval of the mean. *X*-axis—value along the projection image of the first mode of connectivity (g1; from inferior to superior positions); *Y*-axis—value along the projection image of the third mode of connectivity (g3; from anterior to posterior positions).

The connectivity gradients described above were created based on each surface vertex’s connectivity with the ipsilateral hemisphere. To establish which aspects of white matter connectivity were driving each gradient, we created projection images. These indicate for each white matter voxel the weighted average connectivity gradient value of the top three vertices whose streamlines reached that target voxel the most. In other words, we created three volumetric maps showing how each white matter voxel related to each temporal lobe surface connectivity gradient. We then separated these images according to the specific white matter tracts running through the white matter, creating tract projections that allowed us to visualise the contribution of each tract to each gradient. The resulting projection images are shown in Fig. 2, and 2D visualizations of tract projections are shown in Figs. 3 and 4.

A plot showing the relative positions of the white matter tracts of the first and second connectivity modes is shown in Fig. 3. The first connectivity mode, which ran in an inferior–superior direction, was separated into its constituent tracts based on where they project onto the ventral to dorsal axis of the temporal lobe. This ranged from inferior tracts including ILF, IFOF and UF that project to the inferior and middle temporal gyri, with the AF [which mainly projects to the middle and superior temporal gyri (Bajada et al. 2017a)] appearing at an intermediate position along this gradient, to MdLF and AR appearing at very similar positions on the superior end. The second connectivity mode (*y*-axis) separated the AF from all remaining tracts. This suggest that the radial appearance of this gradient on the cortical surface is mostly driven by the unique connectivity profile of the AF. Nevertheless, there seems to exist groups of tracts that lie closer or further away from the AF in this axis. These groups constituted by the ILF and the MdLF (closer), the IFOF and UF (medium separation), and the AR (most separated).

Figure 4 shows the distribution of projection image values of the tracts along the first (*x*-axis) and third (*y*-axis) connectivity gradients. This visualization follows the tract organisation in physical MNI space to a large extent. As mentioned before, the first gradient resembles the ventral–dorsal organisation of the tracts. The third gradient resembles the anterior–posterior distribution, with the UF projecting to more anterior parts of temporal cortex compared to all other tracts. A noticeable larger variability of the tract projection values is noticeable on the *y*-axis (wider confidence intervals—mimicking the effects seen on the ICC of the gradients—Table 1), reflecting the loss in variance explained as we go from lower dimensional gradients to higher dimensional ones. Nevertheless, the gradient still separates tracts present in the anterior temporal lobe (ILF, IFOF,UF) from the tracts only present in its posterior portion (AF, MdLF, AR).

### Functional decoding links connectivity gradients to the functional multiplicity of the temporal lobe

Thus far, we have used imaging of structural connectivity to demonstrate three overlapping connectivity gradients in the temporal lobe and have related these to the specific combination of projections from the major white matter tracts. However, an important step in understanding the organisation of the temporal lobe is to link these anatomical results to function. Therefore, we investigated the relationship of the connectivity gradients with brain activation patterns obtained from the meta-analysis database Neurovault (Gorgolewski et al. 2015). Each group connectivity gradient map was divided into deciles and each decile map was then compared to brain activation maps in Neurovault. This allowed us to ‘decode’ how each aspect of a connectivity map related to task brain activation.

**Fig. 5:**
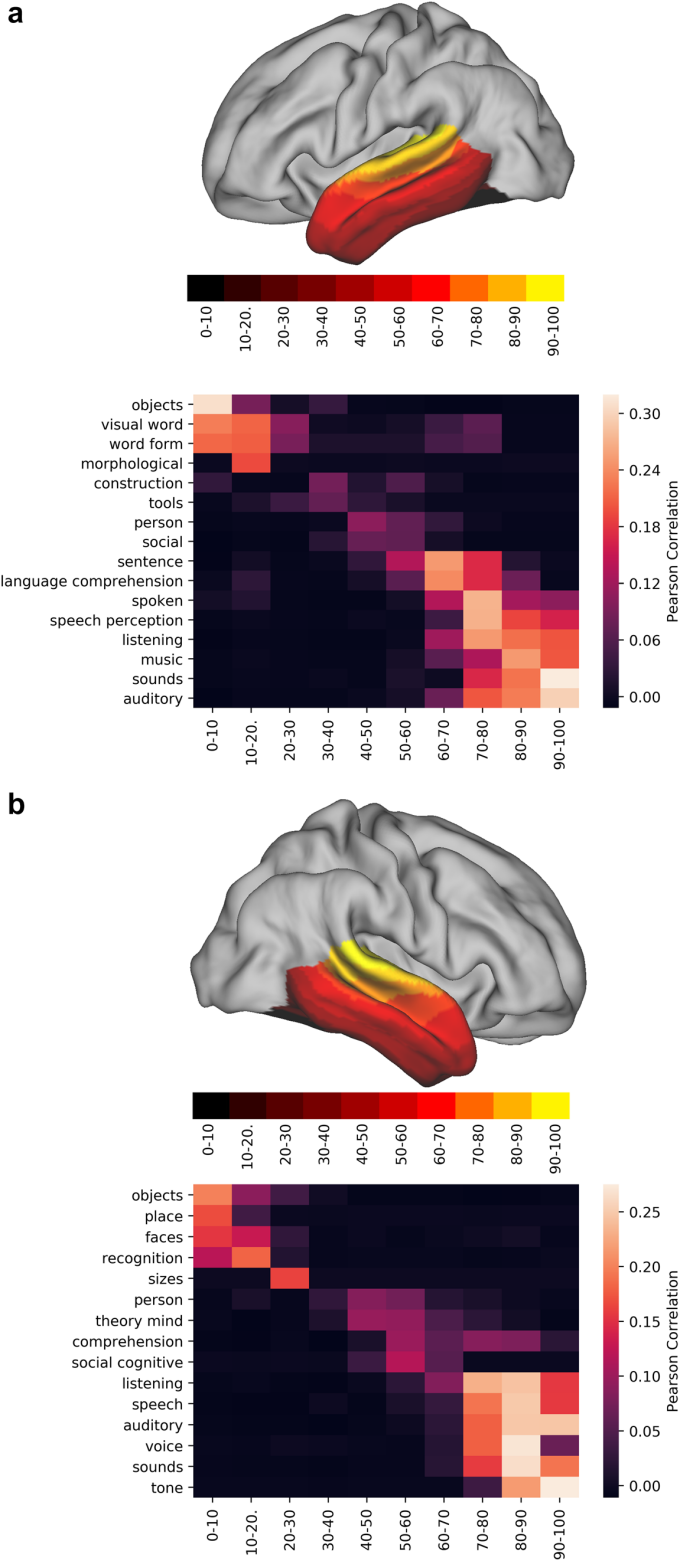
Functional decoding tables of the dominant connectivity gradient of the left (**a**) and right (**b**) temporal lobe. On top of each functional decoding table, the gradient deciles used can be seen separated on a mid-thickness surface. On the table, the *X*-axis represents the decile being decoded and the *Y*-axis depicts the terms that Neurovault identified as most spatially correlated with the connectivity gradient. Values represent Pearson’s spatial correlation of the decile with the whole brain activation pattern associated with the term

Figure 5 shows the functional decoding of dominant connectivity mode on each hemisphere. Above, we showed that the first connectivity gradient in each hemisphere is a ventral to dorsal gradient (Fig. 1a), separating white matter tracts by their anatomical positioning in this axis within the temporal lobe (Fig. 3). Figure 5 indicates that this principle of organisation is accompanied by a gradient of functional roles ranging from visual tasks (objects, place, faces, recognition, sizes, visual word, word form, morphological) at the lower end of its representative connectivity gradient (g1), to auditory tasks (music, sound, auditory, tone) at its higher end. Intermediate values are driven by social tasks (social, person, theory mind, social cognitive) and language tasks, although the extent differs between the hemispheres. The right hemisphere patterns correlate, among others, with tasks such as theory of mind and comprehension (in a general sense—ranging from social cues such as smiles to complex semantic tasks such as narrative comprehension), whereas the left hemisphere shows language comprehension as one of its matching patterns.

Overall, these results suggest that the dominant mode of connectivity in the temporal lobe is anchored by two primary functional roles (vision on the lower end and auditory on the higher end) with multimodal functional roles (social, language—left hemisphere only) represented in intermediate areas.

**Fig. 6:**
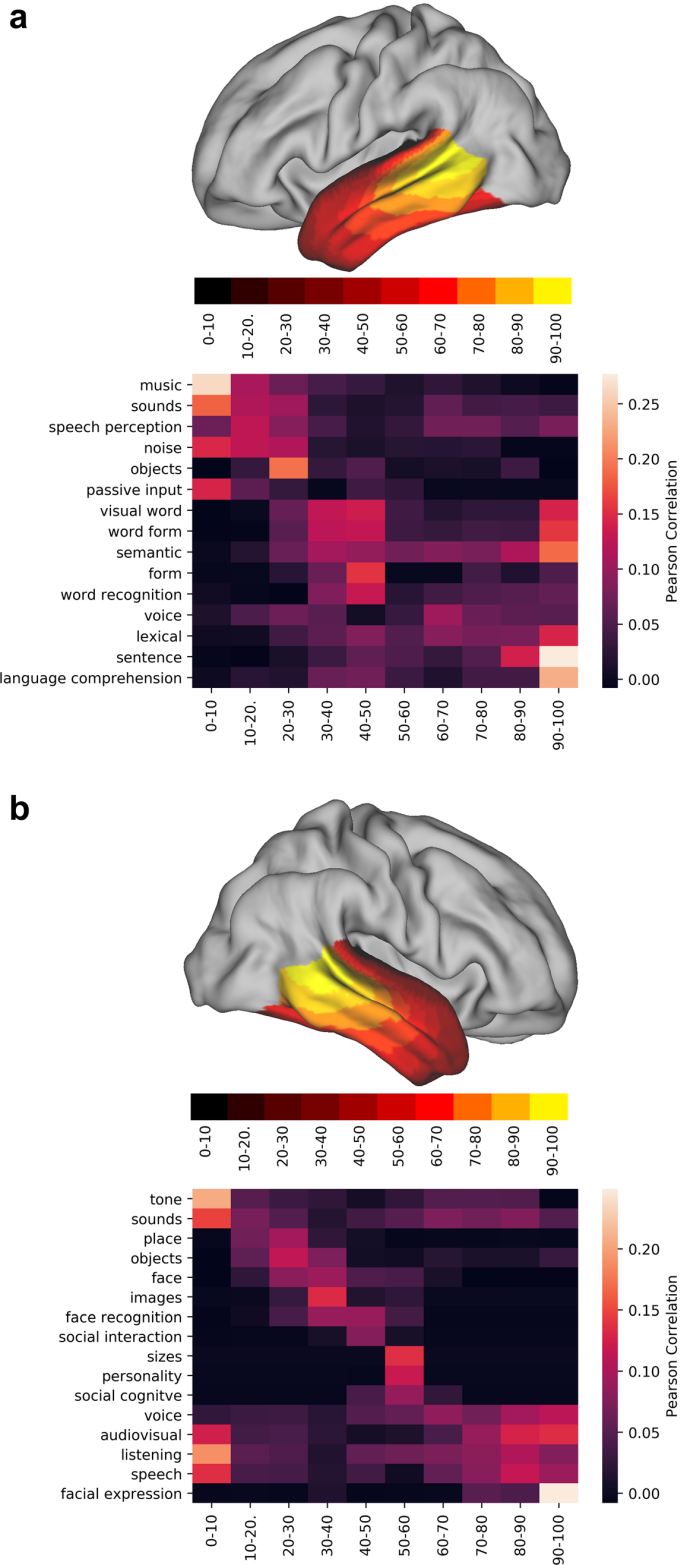
Functional decoding of the second dominant connectivity gradient of the left (**a**) and right (**b**) temporal lobes. On top of each functional decoding table, the gradient deciles used can be seen separated on a mid-thickness surface. On the table, the *X*-axis represents the decile being decoded and the *Y*-axis depicts the terms that Neurovault identified as most spatially correlated with the connectivity gradient. Values represent Pearson’s spatial correlation of the decile with the whole brain activation pattern associated with the term

When analysing projection images such as Fig. 2b, one can tend to interpret the second mode of connectivity as one of ‘arcuate vs all else’. The tract projections in Fig. 3 already paint a different scene, with tracts such as the ILF and the MdLF located closer to the connectivity space of the AF than, for example, AR. The correlation of the deciles of this connectivity gradient complements this picture (Fig. 6). In the left hemisphere, the lower end of the gradient has the highest spatial correlation with acoustic tasks (music, sound, speech perception, noise), then visual (visual word, word form) takes over followed by semantic tasks which present a bimodal overlap pattern, indicating overlap with general language related tasks. The final end is dominated by terms that are associated with different components of language production and comprehension (lexical, sentence, speech comprehension). The right hemisphere is similar to the left hemisphere, but with two key differences: here, the bimodal peak of spatial correlation appears in audiovisual and auditory terms and the higher end of the gradient is maximally correlated with the ‘facial expression’ term instead of language related terms.

In short, the second dominant connectivity mode can be described as ranging from auditory tasks on its lower end, visual tasks in its centre and comprehension tasks on its higher end. In the left hemisphere, these comprehension tasks are related to language (semantics, comprehension, lexical) whereas in the right hemisphere they are related to comprehension of social cues (facial expressions).

**Fig. 7:**
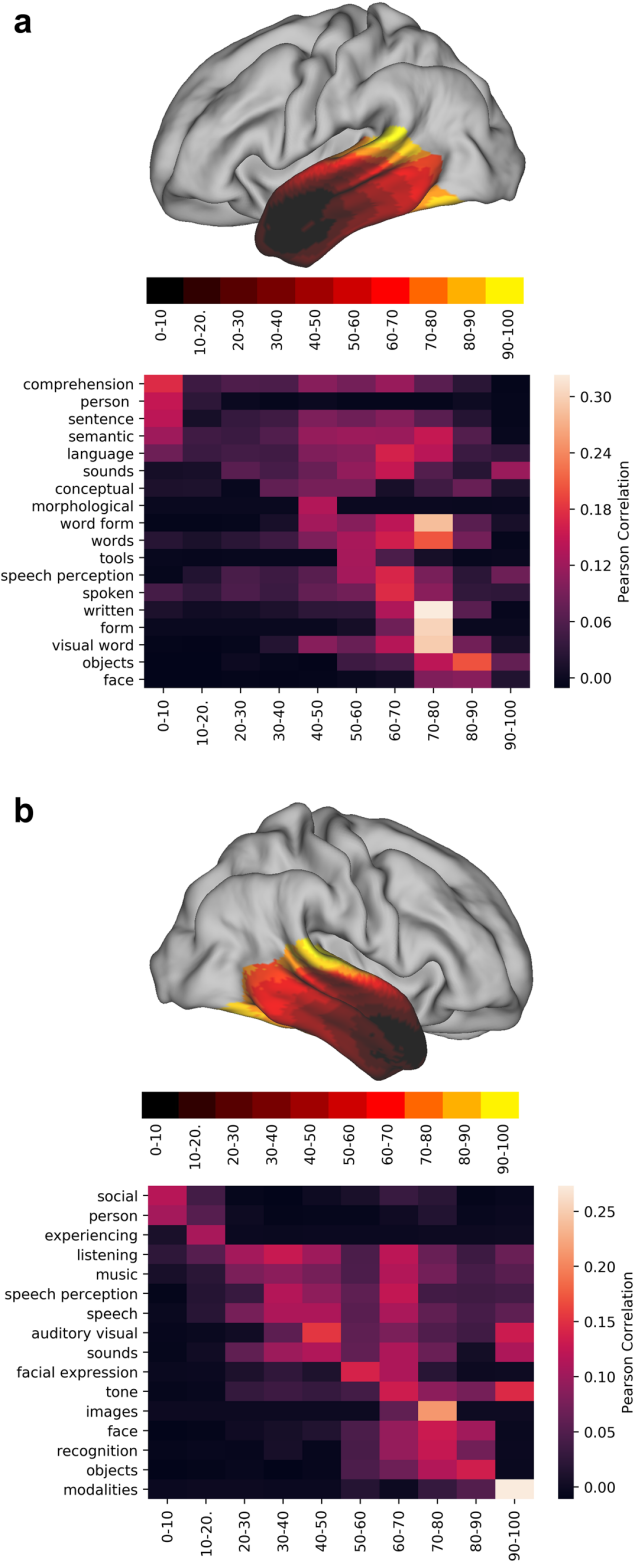
Functional decoding of the second dominant connectivity gradient of the left (**a**) and right (**b**) temporal lobe. *X*-axis represents the decile being decoded and the *Y*-axis depicts the terms that Neurovault identified as most spatially correlated with the connectivity gradient. Values represent Pearson’s spatial correlation of the decile with the whole brain activation pattern associated with the term

The third mode of connectivity (Figs. 1a and 2) appeared to show an anterior–posterior orientation with tract projection images (Fig. 4) confirming that claim. Here, the lower end (anterior end) correlates with different terms in each hemisphere but is dominated by terms associated with tasks that have similar, task general, activation patterns (Fig. 7). Again, language tasks dominate in the left hemisphere (semantic, sentence, comprehension), whereas in the right hemisphere social tasks are predominant (theory of mind, social), although this lateralisation is by no means complete. The higher end on either hemisphere is dominated by what appears to be task specific, with terms corresponding to tasks that have localised activation patterns (face, objects, modalities—multisensory stimuli, visual word, written, spoken). Interestingly, the separation between task-specific and task-general components is much sharper in the right hemisphere than in the left hemisphere, where language-related tasks tend to ‘invade’ posteriorly.

Thus, the third dominant gradient of connectivity distinguishes task specific from task general regions. In the left hemisphere, the task general (anterior end) corresponds to language functions, whereas in the right hemisphere, these correspond to social tasks such as theory of mind tasks. The posterior end of this gradient of connectivity is involved in specific tasks (related to either the auditory or visual domain).

## Discussion

When trying to understand the relationship between brain structure and brain function, it is helpful to understand the underlying principles of organisation of a part of the brain. Here, we attempted to find such principles of organisation for the association cortex of the temporal lobe. We found evidence for three overlapping fine-grained white-matter modes of organisation, formed by gradual shifts of connectivity driven by specific tracts, mapping on to distinct modes of function.

The first gradient of connectivity is chiefly organised along an inferior–superior axis. It is driven by tracts belonging to the ventral visual stream, most prominently the inferior longitudinal fascicle, at its lower end, and the middle longitudinal fascicle and acoustic radiation at its top end. This structural dissociation is echoed in the clear functional dissociation in visual and auditory tasks associated with the two ends of the gradient. These results are in complete agreement with previous studies assigning key roles to the ILF (Herbet et al. 2018) in vision-related tasks and the involvement of MdLF (Wang et al. 2013) and the acoustic radiation (Maffei et al. 2019) in the auditory pathway. Similar inferior–superior gradients have been found when looking specifically at the anterior temporal lobe (ATL) with its inferior end showing peak activations for semantic tasks using picture stimuli, and its superior end being active in semantic tasks using verbal stimuli (Visser et al. 2010). This suggests an inferior–superior gradient in the ATL driven by variations in connectivity to the modality-specific regions of the posterior temporal lobe, which is corroborated by the dependency of this connectivity mode on the position of longitudinal tracts along its inferior–superior axis. The middle part of the gradient reflects the more multimodal nature of the temporal lobe, as evidenced by the higher level tasks associated with it and the connections that reaches all the way to the prefrontal cortex through the inferior fronto-occipital fascicle. This is consistent with suggestions that this tract reaches multi-modal temporal regions in parts of the temporal cortex that have expanded in the hominin lineage (Bryant et al. 2019). In summary, the first gradient of organization of the temporal lobe highlights the pre-eminence of longitudinal tracts in its white matter composition, with a clear separation between ventral and dorsal tracts accompanied by substantial functional segregation.

The second gradient radiates outwards anteriorly from the middle posterior temporal gyrus. This is largely driven by the arcuate fascicle in both hemispheres, but the subsequent radiation also showed a subtle distinction between the various association tracts. Functionally, the maximum values of the connectivity gradient correlate highly with language-related terms (lexical, sentence, language comprehension) consistent with the dominance here of the arcuate fascicle that connects the pMTG to the inferior frontal gyrus (ITG). Additionally, the terms ‘semantic’, ‘visual word’ and ‘word form’ were highly correlated with the higher end of this connectivity gradient, possibly reflecting the role of the arcuate fascicle in mediating communication between the posterior temporal and inferior frontal cortex, particularly on the left hemisphere (Davey et al. 2016; Gonzalez Alam et al. 2019). In the right hemisphere, language terms are intermixed with social terms, with facial expression showing the highest correlation pattern. Functional correlations with intermediate gradient levels also show a hemispheric difference, with terms related to word processing in the left hemisphere and terms related to social information processing in the right hemisphere. Interestingly, some of the tracts driving the intermediate-level gradient values have been associated with both these processes (Catani and Bambini 2014; Lai and Reilly 2015; Mars et al. 2016; Noonan et al. 2018). The functional correlations of the lowest values along the gradient were dominated by auditory terms bilaterally on its lower end, consistent with the lower tract projection of the acoustic radiation on this connectivity gradient. Concisely, the second dominant connectivity mode can be described as ranging from auditory tasks on its lower end (involved with the AR), and comprehension tasks on its higher end (enabled by the AF, ILF and MdLF).

The third gradient and its subsequent brain activation profile can be described as reflecting higher vs lower order information processing, which dovetails with the long-held hierarchical models of association cortex organisation (Fuster 2004). In the temporal lobe, this idea is based on observations that as you move anteriorly in temporal association cortex, cortical areas respond to increasingly larger parts of the visual field, and appear to process increasingly abstract information increases in the antero-posterior axis (Kravitz et al. 2013). It is also consistent with the more recent view of the anterior temporal cortex as a hub for high-level semantic cognition (Lambon Ralph et al. 2010) and the overall task-general to task-specific organisation in the temporal lobe (Bajada et al. 2017b; Jackson et al. 2018). Interestingly, there is a noticeable functional dissociation of semantic tasks on the left hemisphere (as in the second gradient), with the term ‘semantic’ appearing in the most highly correlated terms at its lower end reflecting the multimodal semantic representation in the anterior temporal lobe and again at intermediate values—now associated with the specialized role of the pMTG in semantic control (Noonan et al. 2013). This dual representation of the term ‘semantic’ at different points along the third gradient reflects two different domains of semantic tasks and gives further evidence for a task-general to task-specific gradient in the human temporal lobe. Therefore, the third dominant gradient of connectivity ranges from task general regions involved in combination of stimuli to task specific regions involved in conduction of stimuli. In the left hemisphere, the task general (anterior end) plays an important role in multimodal semantic processing, while in the right hemisphere, these areas are active in social tasks such as theory of mind tasks. On the posterior end of this gradient, we find regions that activate in a narrow range of stimuli-specific tasks related to either the auditory, visual or semantic control.

From a biological validity standpoint, it is not unreasonable to envision the white matter connectome of a given area as superposition of different components (or ‘modes’ as we have used throughout this manuscript), each explaining some attribute of the connectivity fingerprint and related to a functional dimension. But, while we have described and discussed gradients of connectivity as representative of different organizational principles of the temporal lobe, there is an important orthogonality constraint, resulting from the algorithm choice (Laplacian eigenmaps) imposed on these gradients. In other words, while the first gradient is fully data driven, all subsequent gradients will explain less variance while being orthogonal (but not independent) to each other. The combination of these two factors (sequential loss of degrees of freedom and decrease in variance explained) might explain the decrease in ICC (Table 1) seen when moving from the first gradient to the higher order gradients that had already been seen in previous work (Haak et al. 2018) reinforcing the need for dimensionality estimation prior to dimensionality reduction.

In general, tract projections did not seem to indicate significant lateralisation effects. This finding agrees with that of Bajada and co-workers (2019), who observed that despite the fact that connectivity strengths might differ between hemispheres, the overall spatial pattern of connections is very similar. It suggests that Laplacian eigenmaps uncover a unique aspect of connectivity and is complementary to more traditional analyses of physical characteristics of fibre bundles like streamline count or cross section (Raffelt et al. 2017). The link of connectivity gradient patterns with brain activation maps indicates a tendency for right hemisphere activation in social tasks and left hemisphere activation for language and semantic tasks. This dissociation is consistent with observations in the functional neuroimaging literature (Hurley et al. 2015; Olson et al. 2013; Pobric et al. 2016; Rice et al. 2015). Yet, when mapping major white-matter tracts on our gradients, we found a strikingly similar pattern across hemispheres. Thus, the crucial information our study adds is the observation that even when distinguishing between distinct principles of structural connectivity organisation, there is no significant hemispheric difference between their composing tracts. Therefore, a similar overall anatomical infrastructure, both in terms of the order and type of gradients and of tracts driving these gradients, supports quite different aspects of cognition across the two hemispheres.

Previous studies have also looked at gradients of connectivity in the temporal lobe, but our results differ in some important ways. Binney and colleagues (2012) described a non-graded major dissociation between rostral, mid and caudal subregions of the temporal lobe regarding their connectivity to prefrontal and parietal cortices. In contrast, our results suggest that the principal direction of connectivity change is along the superior–inferior axis. This apparent discrepancy in results can be explained by the inclusion of the occipital cortex in our analysis since the lower end of this connectivity mode is occupied by tracts connecting the temporal and occipital lobes (ILF and IFOF). Visser and colleagues (2012) described two principle axes of convergence of information in the temporal lobe: one lateral, towards MTG, and the other longitudinal, towards the ATL. While we observe similar patterns of gradual change (lateral and longitudinal gradients), they are now driven by structural connectivity differences instead of gradual changes in activation patterns given a semantic task. In another study, Bajada and colleagues (2017b) used spectral reordering on diffusion tractography of the temporal lobe to uncover its principles of organisation, obtaining two main directions of connectivity change. One direction was medial to lateral, and the second was an anteroventral to posterodorsal along the lateral surface. In contrast to Bajada and colleagues, we did not include the medial temporal lobe in our region of interest, as this would group together two very different types of cortex. In particular, the inclusion of the hippocampus is likely to change the results quite substantially. This can be illustrated by the tract projection plot in Fig. 4, where the non-inclusion of the second gradient caused the arcuate to lie to be in an intermediate region of the ventral–dorsal axis and on the posterior end of the anterior posterior axis. Despite these conflicting results, the authors of this previous study also state that ‘the principal axis of organisation along the lateral surface may reflect connectivity changes associated with the relative functional specialisation of the dorsal and ventral pathways’ on the left hemisphere and that ‘the axis of organisation along the lateral surface seen in the right hemisphere may reflect the division between ventral (lexico-semantic) processes and dorsal spatial processes’ which is in agreement with our findings. Recently, Bajada and colleagues (2019) revisited the issue of temporal lobe organisation, this time using Laplacian eigenmapping. They also found the ideal dimensionality to be three and replicated their findings on gradual connectivity change patterns, with the added dimensionality enabling them to propose the anterior temporal lobe as a convergence zone, equidistant from all extremes of the connectivity space. This agrees with our results as the removal of the hippocampus from the analysis shifts the anterior temporal lobe to the edge of the third connectivity gradient.

Our results are subject to certain limitations. First, there are shortcomings inherent to diffusion tractography, especially when trying to map precise end-to-end connectivity (Jbabdi et al. 2013). However, our results are less susceptible to false positives and false negatives coming from probabilistic tractography since the input data to the Laplacian eigenmap algorithm is based on connectivity similarity between vertices (that depend on their whole hemisphere connectivity fingerprint) rather than relying on precise description of specific fibre bundles. In other words, projection images and tract projections are generated by the connectivity gradients, not the other way around. Second, the functional decoding of gradients could be extended in future work as the currently analysed metric is a simple Pearson correlation between the binarized decile gradient maps and the images associated with certain terms in the Neurosynth database. Factors such as ambiguity of the terms, and few non-zero values in the analysed deciles might influence the results (later editions of the Neurovault pipeline will account better for maps that do not cover the whole brain). Future work is needed to investigate the reported gradients and task fMRI data within the same subjects to more precisely define the structure–function relationship and to investigate the covariance of the two across individuals.

Having established the principles of organisation of the human temporal cortex, a crucial next step will be to investigate how these differ in other species. Similar temporal cortex tracts have been reported in humans, great apes, and macaques (Bryant et al. 2019; Makris and Pandya 2008; Mars et al. 2019; Schmahmann and Pandya 2006). The human temporal lobe, however, supports a number of functions that are thought to be uniquely elaborated in humans, and it has been suggested that temporal lobe connectivity has undergone substantial reorganisation to support these unique abilities (Eichert et al. 2019; Mars et al. 2013; Patel et al. 2019). The extension of the arcuate fascicle into the middle temporal gyrus is the most prominent example (Eichert et al. 2018; Rilling et al. 2008), but more subtle changes in, for instance, the ventral visual stream have also been reported (Bryant et al. 2019; Latini et al. 2017; Roumazeilles et al. 2018). The gradients described here provide a data-driven measure by which different species’ brain organisation can be directly and quantitatively compared. This will be the focus of ongoing research.

## Electronic supplementary material

Below is the link to the electronic supplementary material.

Supplementary file1 (DOCX 3498 kb)
