# Supplementary material for: Principles of temporal association cortex organisation as revealed by connectivity gradients
Source: Brain Struct Funct. 2020 Mar 10;225(4):1245–60. doi: 10.1007/s00429-020-02047-0 (PMC7270054; doi:10.1007/s00429-020-02047-0)
Supplement: Supplementary file 1 — Supplementary file1 (DOCX 3498 kb) [file 429_2020_2047_MOESM1_ESM.docx]

Supplementary Figures


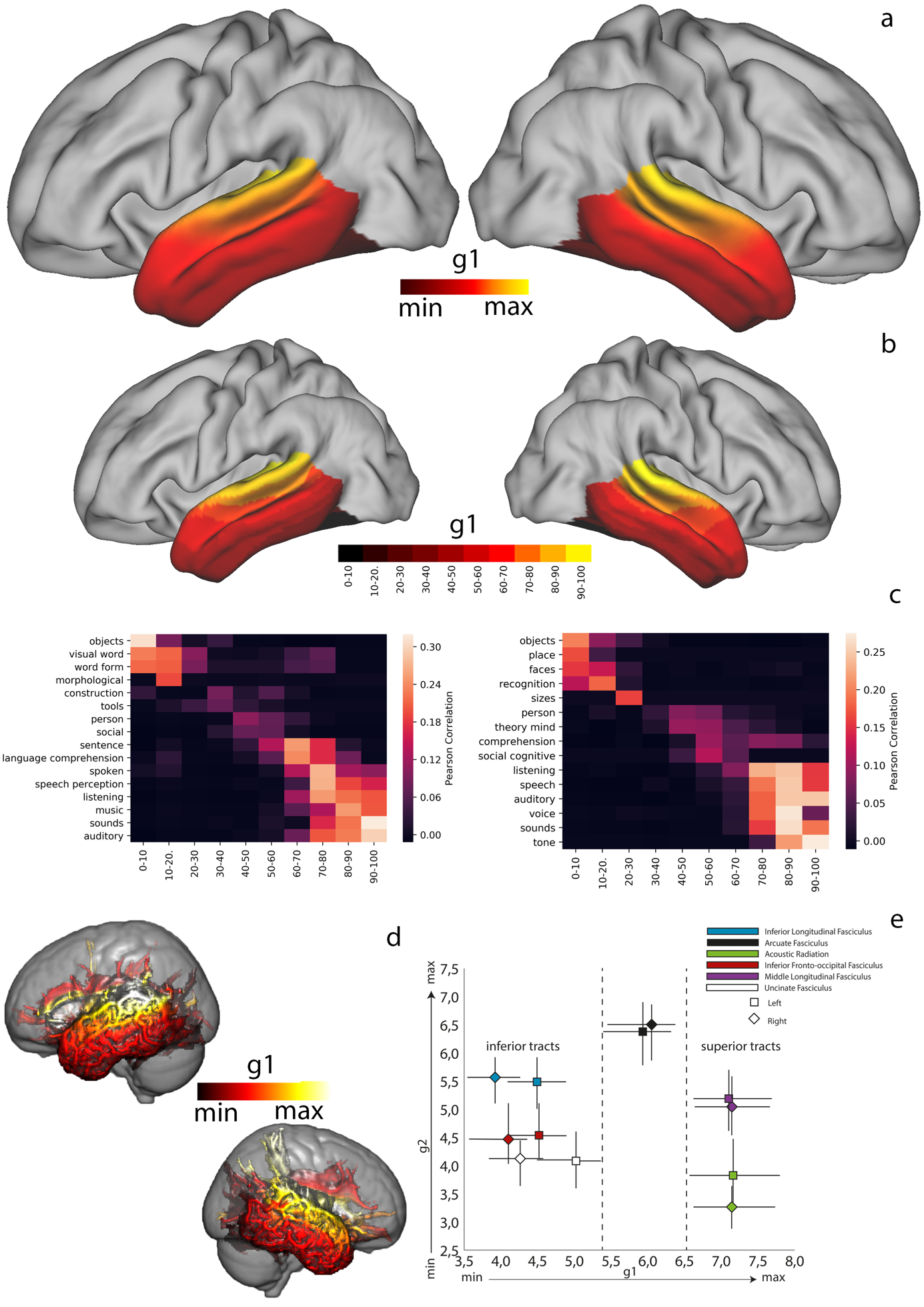


Supplementary Figure 1 – Full overview of the entire analysis pipeline results for the dominant connectivity mode. (a) Group averaged dominant mode of connectivity (g1) projected on a midthickness surface. (b) Group averaged dominant mode of connectivity (g1) deciles used for functional decoding in Neurosynth. (c) Functional decoding of the dominant connectivity gradient. (d) Projection map of the dominant mode of connectivity (g1) of a representative subject – 103818. (e) Projection image average value of the temporal lobe’s relevant tract projections. Dashes represent the bootstrapped 95% confidence interval of the mean. X axis - Value along the projection image of g1 (from interior to superior positions); Y axis - Value along the projection image of g2 (radiates from the AF).


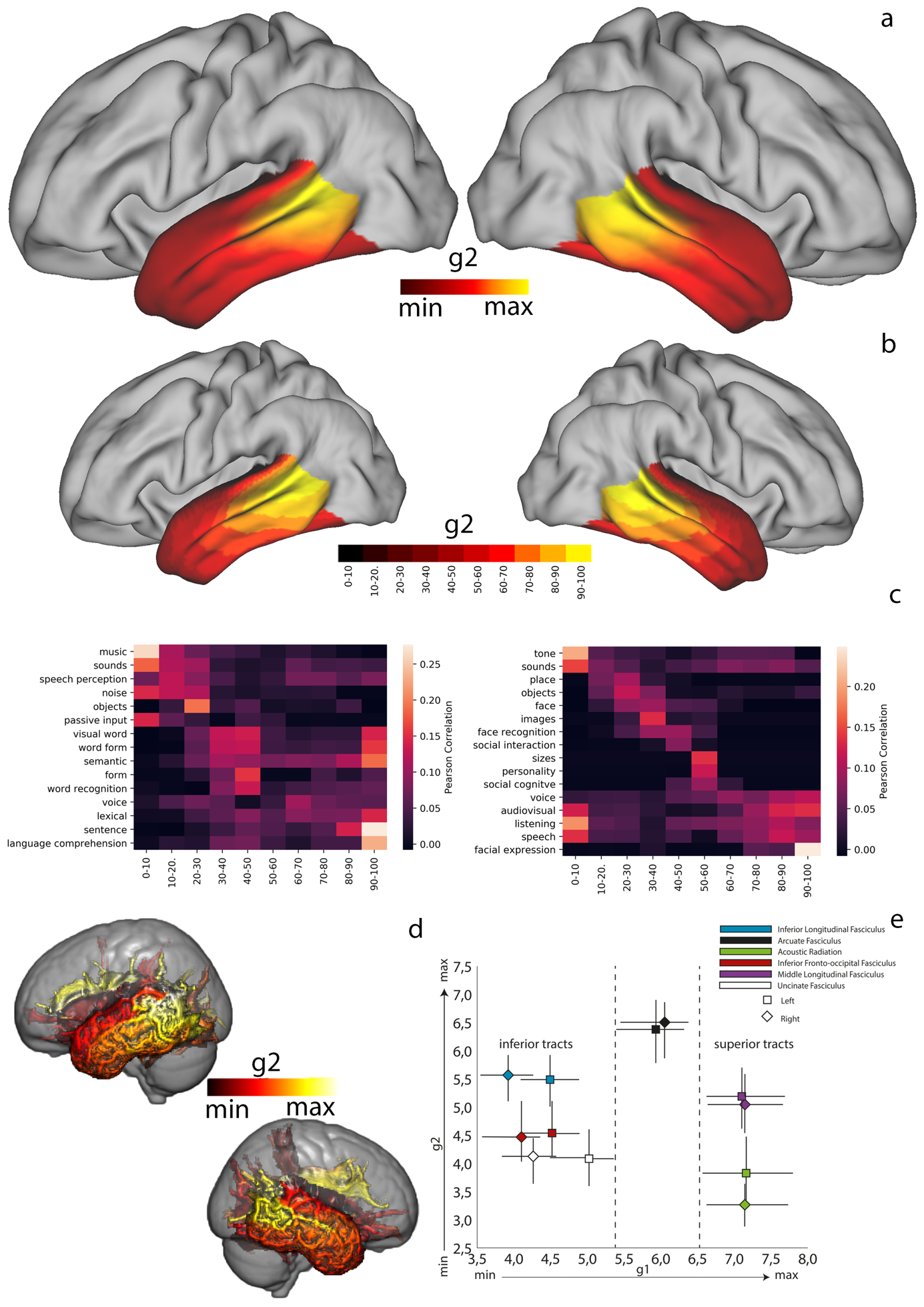


Supplementary Figure 2 – Full overview of the entire analysis pipeline results for the second dominant connectivity mode. (a) Group averaged second dominant mode of connectivity (g2) projected on a midthickness surface. (b) Group averaged second dominant mode of connectivity (g2) deciles used for functional decoding in Neurosynth. (c) Functional decoding of the second dominant connectivity gradient. (d) Projection map of the second dominant mode of connectivity (g2) of a representative subject – 103818. (e) Projection image average value of the temporal lobe’s relevant tract projections. Dashes represent the bootstrapped 95% confidence interval of the mean. X axis - Value along the projection image of g1 (from interior to superior positions); Y axis - Value along the projection image of g2 (radiates from the AF).


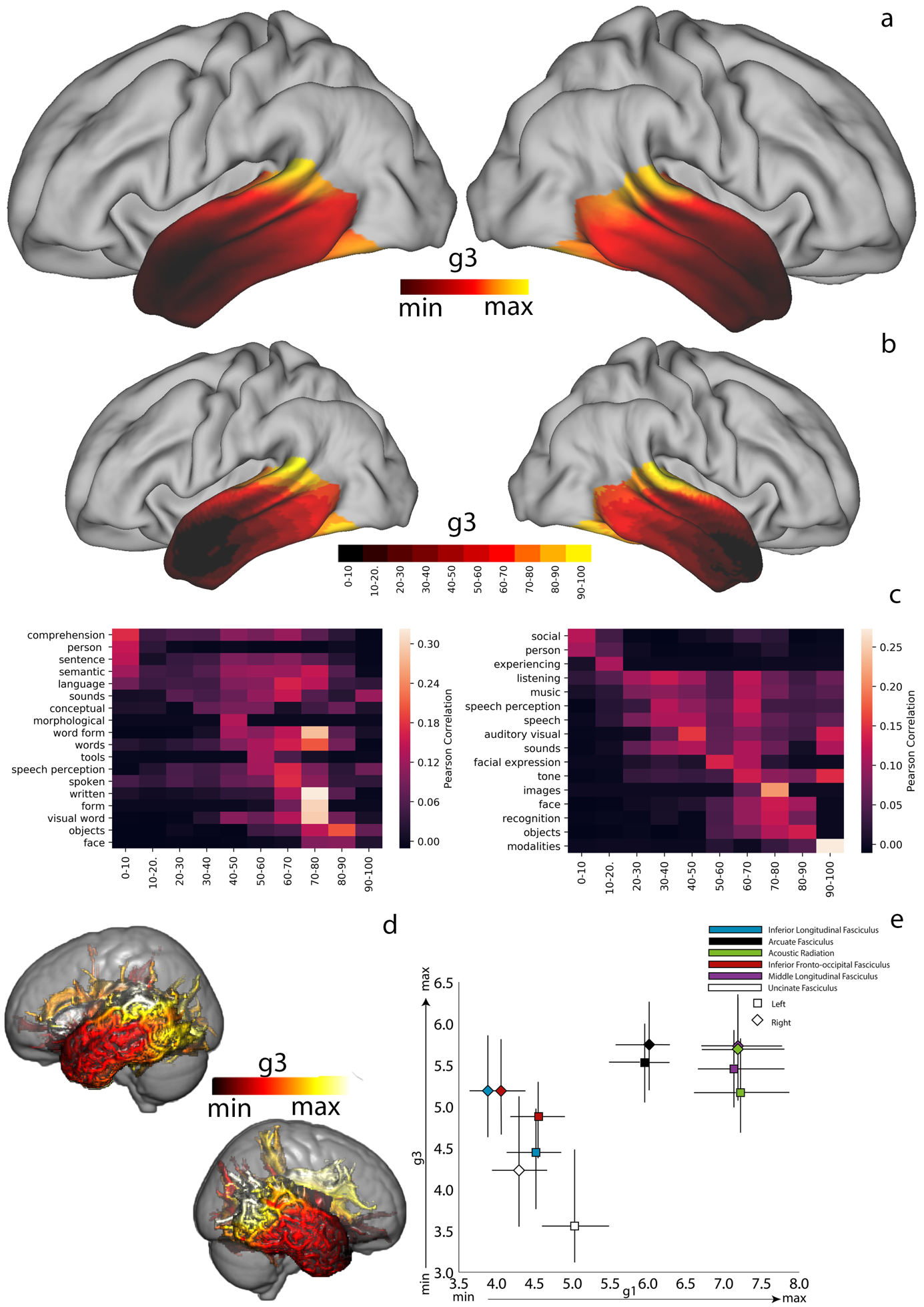


Supplementary Figure 3 – Full overview of the entire analysis pipeline results for the third dominant connectivity mode. (a) Group averaged third dominant mode of connectivity (g3) projected on a midthickness surface. (b) Group averaged third dominant mode of connectivity (g3) deciles used for functional decoding in Neurosynth. (c) Functional decoding of the third dominant connectivity gradient. (d) Projection map of the third dominant mode of connectivity (g2) of a representative subject – 103818. (e) Projection image average value of the temporal lobe’s relevant tract projections. Dashes represent the bootstrapped 95% confidence interval of the mean. X axis - Value along the projection image of g1 (from interior to superior positions); Y axis - Value along the projection image of g3 (from anterior to posterior positions).
